# Trans-Renal Cell-Free Tumor DNA for Urine-Based Liquid Biopsy of Cancer

**DOI:** 10.3389/fgene.2022.879108

**Published:** 2022-04-27

**Authors:** Sarah M. Dermody, Chandan Bhambhani, Paul L. Swiecicki, J. Chad Brenner, Muneesh Tewari

**Affiliations:** ^1^ Department of Otolaryngology-Head and Neck Surgery, University of Michigan Health System, Ann Arbor, MI, United States; ^2^ Department of Internal Medicine, Division of Hematology/Oncology, University of Michigan Medical School, Ann Arbor, MI, United States; ^3^ Veterans Affairs Ann Arbor Healthcare System, Ann Arbor, MI, United States; ^4^ Department of Biomedical Engineering, University of Michigan, Ann Arbor, MI, United States; ^5^ Center for Computational Biology and Bioinformatics, University of Michigan, Ann Arbor, MI, United States

**Keywords:** trans-renal, liquid biopsy, cell-free DNA, circulating tumor DNA, ctDNA, urine, biomarker, cancer

## Abstract

Cancer biomarkers are a promising tool for cancer detection, personalization of therapy, and monitoring of treatment response or recurrence. “Liquid biopsy” commonly refers to minimally invasive or non-invasive sampling of a bodily fluid (i.e., blood, urine, saliva) for detection of cancer biomarkers such as circulating tumor cells or cell-free tumor DNA (ctDNA). These methods offer a means to collect frequent tumor assessments without needing surgical biopsies. Despite much progress with blood-based liquid biopsy approaches, there are limitations—including the limited amount of blood that can be drawn from a person and challenges with collecting blood samples at frequent intervals to capture ctDNA biomarker kinetics. These limitations are important because ctDNA is present at extremely low levels in plasma and there is evidence that measuring ctDNA biomarker kinetics over time can be useful for clinical prediction. Additionally, blood-based assays require access to trained phlebotomists and often a trip to a healthcare facility. In contrast, urine is a body fluid that can be self-collected from a patient’s home, at frequent intervals, and mailed to a laboratory for analysis. Multiple reports indicate that fragments of ctDNA pass from the bloodstream through the kidney’s glomerular filtration system into the urine, where they are known as trans-renal ctDNA (TR-ctDNA). Accumulating studies indicate that the limitations of blood based ctDNA approaches for cancer can be overcome by measuring TR-ctDNA. Here, we review current knowledge about TR-ctDNA in urine as a cancer biomarker approach, and discuss its clinical potential and open questions in this research field.

## Introduction

Cancer biomarkers have emerged as promising tools of precision medicine for designing effective treatment regimens, evaluating treatment response, and detecting primary or recurrent cancers. The sampling of body fluid to detect cancer cells or material derived from cancer cells is commonly referred to as “liquid biopsy” (Mader and Pantel 2017). Most commonly, liquid biopsy involves the detection and analysis of circulating tumor cells (CTC), or of fragments of cell-free circulating tumor DNA (ctDNA) that are shed by dying cancer cells into the bloodstream. This offers a minimally-invasive tool to detect and gather information on a patient’s cancer, including characterization of potential tumor genetic heterogeneity, assessment of treatment response, or even emergence of treatment resistance mechanisms (e.g., via detection of specific therapeutic resistance mutations) (Alix-Panabières and Pantel 2021).

Although blood-based liquid biopsy has been clinically successful across many cancer types, this approach still faces limitations, including the limited quantity of blood that can be obtained from a patient for analysis, challenges with obtaining blood samples at high frequency for serial monitoring, and difficulties with reaching patients who have limited access to clinical facilities for phlebotomy. This is relevant because ctDNA is present at extremely low levels in plasma and is undetectable using standard blood sampling volumes in many cancer patients. In addition, there is evidence that measuring ctDNA biomarker kinetics at high time resolution can be useful for clinical prediction, and there is a great need to enable equitable access to healthcare for all members of society.

**FIGURE 1:**
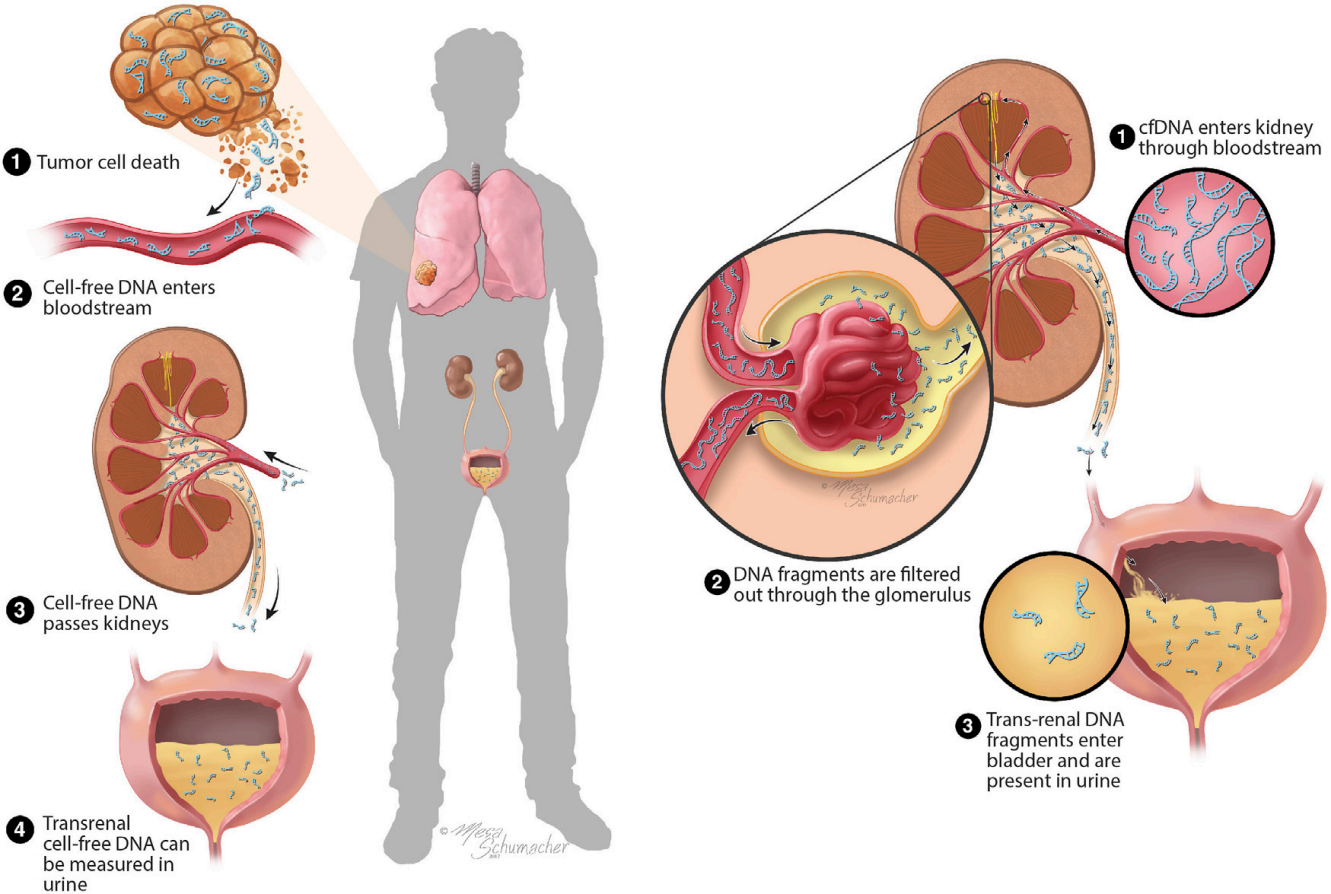
Current model of formation of trans-renal ctDNA. The drawing on the left provides a conceptualized overview of the process of ctDNA generation and its transit to the urine. The drawing on the right provides a more detailed conceptualization of the presumed transit of cell-free DNA through the glomerular barrier of the kidney. Abbreviation used in the drawing: cfDNA, cell-free DNA. The artwork in this figure was created by Mesa Schumacher, M.A.

An alternative approach that has been gaining momentum in the cancer research field is the use of urine as the biofluid specimen for liquid biopsy, in particular for cancers present in organs across the body, rather than just those originating in the urinary tract (e.g., bladder cancer). Whereas cancers present in the urinary tract can directly shed cancer cells or tumor DNA into the urine, there is a phenomenon known as “trans-renal” transit of DNA that enables urine-based access to ctDNA for cancers from distant organs. In this process, fragments of cell-free DNA (cfDNA) present in blood (including ctDNA fragments) are filtered through the kidney’s glomerular filtration system, into the urine (Wang et al. 2017; Moreira et al. 2009; Crisafulli et al. 2019; Lu and Li 2017) (Figure 1). The resulting ctDNA fragments present in the urine are known as trans-renal ctDNA (TR-ctDNA). A key feature of TR-ctDNA is that it allows, in principle, the detection of ctDNA released from cancer in virtually any organ in the body, as long as ctDNA is present in the plasma and able to transit into the urine.

Thus, the phenomenon of trans-renal passage of ctDNA presents an exciting emerging approach for completely non-invasive liquid biopsy with broad potential applications. In this review, we will discuss what is known about the biology of trans-renal DNA, studies to date on TR-ctDNA as a cancer biomarker, its future clinical potential and current gaps in knowledge.

## Non-Oncology Studies Establishing the Trans-renal Cell-Free DNA Concept

A seminal study examining the trans-renal cell-free DNA concept was conducted by Botezatu *et al.* and marks the first demonstration of trans-renal DNA in both animal and human models (Botezatu et al. 2000). The authors sought to assess if cfDNA from the bloodstream crosses the glomerular filtration barrier and can be analyzed via standard genetic techniques. This group examined both human and animal models and described the phenomenon of trans-renal passage of circulating DNA. For the animal experiments, mice were injected with either human Raji cells (a lymphoblast-like human cell line) or radiolabeled DNA. Analysis of urine from the mice injected with human Raji cells demonstrated that human genomic *Alu* sequences were detectable in the urine. The injected radiolabeled DNA was also detectable in urine in a polymeric form, albeit this represented only a small fraction (∼0.06%) of the total injected DNA (Botezatu et al. 2000). When examining urine of humans receiving blood transfusions, Botezatu *et al.* found male-specific DNA sequences in females who had received blood transfusion from a male donor, which were presumed to represent DNA that had been present in the transfused blood product and crossed the kidney’s glomerular barrier (Botezatu et al. 2000). Similarly, pregnant females with male fetuses showed detectable male-specific DNA sequences in the mother’s urine (Botezatu et al. 2000).

Although the study of Botezatu *et al.* provided early proof-of-concept that the renal barrier in both mice and humans is permeable to cfDNA in some form, some early subsequent studies cast controversy over this finding, as they reported an inability to detect cfDNA of male fetuses in the urine of pregnant women carrying male fetuses (Y. Li et al., 2003; Illanes et al., 2006), a result that in retrospect may have been due to differences in urine processing and preservation or other technical differences. This is presumed to be the case because multiple other follow-up studies have confirmed the presence of trans-renal DNA in diverse clinical settings, including in pregnancy (Tsui et al., 2012; Shekhtman et al., 2009; Koide et al., 2005; S. C. Y. Yu et al., 2013), hematopoietic stem cell transplantation (Cheng et al., 2017; Hung et al., 2009), infectious disease (Cannas et al., 2008), and systemic histiocytic disorders (Hyman et al., 2015).

## Trans-Renal Cell-Free DNA in Oncology

Soon after the earliest studies of trans-renal cell-free DNA in pregnancy, multiple groups reported investigations using this approach in the setting of tumor-derived DNA in cancer patients. Some of the earliest studies demonstrated proof-of-concept for TR-ctDNA in the setting of colorectal cancer patients, where mutant K-ras DNA (Su et al., 2004, 2005, 2008) and subsequently hypermethylated vimentin gene sequences (B. P. Song et al., 2012) were detected in urine using PCR analysis. Concomitant gel fractionation experiments indicated that TR-ctDNA fragment length is in the 150–250 bp range (Su et al., 2004).

In parallel work, Chan *et al.* studied TR-ctDNA in EBV-associated nasopharyngeal carcinoma, by assaying the urinary excretion of circulating EBV DNA in relation to plasma EBV DNA levels (Chan et al., 2008). In this form of cancer, the EBV DNA is present in episomal form in the cancer cells, and when released from cells, it represents in effect a form of ctDNA. Using real-time PCR, this group quantified the amount of urine EBV ctDNA of seventy-four patients with nasopharyngeal carcinoma. They found detectable EBV TR-ctDNA in over half of the study cohort and that there was a positive correlation between plasma and urine concentrations of EBV ctDNA. In addition, the authors compared results obtained using 59-bp and 76-bp amplicon assays, which indicated that the EBV sequences found in urine are predominantly <76 bp because of greater signal obtained with the 59 bp assay.

Subsequently, a series of studies focused on detection of EGFR or KRAS mutations as TR-ctDNA in urine of patients with non-small cell lung cancer (Berz et al. 2015; Reckamp et al. 2016; Chen et al. 2017; Tchekmedyian et al. 2017; Husain et al. 2017; Franovic et al. 2017; F. Li et al. 2017; Wang et al. 2017; H. Zhang et al. 2018; Hu et al. 2018; Jin, Gou, and Qian 2019). These studies, some of which used commercial assays available at the time, demonstrated not only that urinary TR-ctDNA is detectable in lung cancer patients, but more specifically that the therapeutic resistance-associated EGFR T790M mutation (Berz et al. 2015; Reckamp et al. 2016; Chen et al. 2017; Franovic et al. 2017; F. Li et al. 2017) could be detected non-invasively through urine. Furthermore, multiple of these reports demonstrated the longitudinal tracking of EGFR mutant TR-ctDNA in non-small cell lung cancer patients over time, including one study that pursued high time-resolution urine collection (i.e., daily collection) and found associations between TR-ctDNA kinetics early during therapy and treatment outcomes (Husain et al. 2017).

More recently, there has been renewed attention to TR-ctDNA in the context of its use as a biomarker for colorectal cancer, with PCR-based assays of targeted mutations (e.g., KRAS, BRAF) (T. Song et al. 2018; H. Yu et al. 2020; Ohta et al. 2021) or methylated DNA loci (Bach et al. 2021) confirming that mutant TR-ctDNA is detectable in the urine of a majority of colorectal cancer patients whose tissue biopsies showed the same mutations. In the Bach et al. study, a combined methylated DNA marker panel was able to detect up to 70% of colorectal cancer patients with 86% specificity (Bach et al. 2021). Furthermore, two recent studies used next-generation sequencing (NGS) to characterize TR-ctDNA in colorectal cancer patients. One study, utilizing whole exome sequencing, found that cancer-specific mutations and copy number changes could be identified in TR-ctDNA and estimated a median length of 112 bp for TR-ctDNA fragments (Crisafulli et al. 2019). The other study utilized targeted NGS to characterize ctDNA in urine, in order to detect minimal residual disease in colorectal cancer patients after neoadjuvant chemotherapy (Pellini et al. 2021). This study also confirmed the detection of TR-ctDNA in a majority, though not all, patients with minimal residual disease after treatment and estimated the average TR-ctDNA length to be 150 bp (Pellini et al. 2021).

One additional next-generation sequencing (NGS) study recently published examined TR-ctDNA in the context of patients with glioma, an aggressive type of brain tumor that is commonly lethal (Mouliere et al. 2021). Using a targeted hybridization-capture NGS technique to look for specific tumor mutations, the investigators found that TR-ctDNA could be detected in six out of eight patients at a pre-surgery urine collection and estimated the median length of the TR-ctDNA fragments to be 101 bp. It is worth noting that some additional NGS- and non-NGS-based studies have detected ctDNA in urine of kidney cancer patients (Smith et al. 2020; Nuzzo et al. 2020; Eisenberger et al. 1999), but will not be discussed further here because it is difficult to know the extent to which such ctDNA represents TR-ctDNA vs. “post-renal” ctDNA that is introduced directly into the urothelial tract through tumor invasion into the renal collecting system.

The TR-ctDNA research field has continued to expand to encompass other types of cancer, including the demonstration of TR-ctDNA detection in patients with breast cancer (Liu and Liu 2018; Guan et al. 2020; J. Zhang, Zhang, and Shen 2020; Zuo et al. 2020), liver cancer (Hann et al. 2017), pancreatic cancer (Terasawa et al. 2019) and gastric cancer (Shi et al. 2017).

## Discussion

The expanding base of published literature investigating TR-ctDNA as a biomarker for diverse cancer types highlights the strong potential of this approach for enabling completely non-invasive cancer diagnostics, using a self-collected biospecimen that does not have the collection frequency or sample volume limitations of blood-based liquid biopsies. However, this is still an emerging area and there are multiple gaps in knowledge that need to be addressed as part of successful development of this approach toward effective clinical application.

One of the important questions pertains to the physical nature of TR-ctDNA, in particular the length of fragments that comprise TR-ctDNA, because this impacts the design of TR-ctDNA assays. Literature to date in cancer studies is conflicting, ranging from 150–250 bp size in early studies, to 100–150 bp from sequencing studies, to <78 bp from a quantitative PCR-based study. Although it is tempting to give more credence to the NGS-based studies, they are also subject to technical biases related to the library preparation methods used, including for example, targeted capture-seq methods, which have inherent fragment length biases. Further analysis using sequencing methods that have minimal bias will be important for determining most accurately the size profile of TR-ctDNA in diverse cancer types. A recent NGS study took a step in this direction by characterizing fragmentation patterns of total cell-free DNA in urine from cancer patients and healthy controls (Markus et al. 2021), which were found to differ and were able to distinguish between these two groups. However, because that study focused on total cfDNA and not TR-ctDNA in particular, it is difficult to know to what extent the fragment sizes observed can be extrapolated to describe TR-ctDNA.

Another class of open questions pertains to pre-analytic variables impacting TR-ctDNA analysis, which are important to characterize and where possible, to control, in order to enable reliable diagnostic tests. One of these pre-analytic variables is ensuring stability of TR-ctDNA at the point of collection. At least two studies of the kinetics of urine cfDNA degradation indicate that cfDNA in urine specimens is not stable, decaying under first-order kinetics with a half-life of 2.6–5.1 h in one study (Cheng et al. 2017), and with a half-life that was too short to accurately measure in the other study (Yao et al. 2016). The addition of EDTA at the point of collection has been reported to stabilize cell-free DNA in urine (Lee et al. 2020; Murugesan et al. 2019; Bosschieter et al. 2018), suggesting that endogenous deoxyribonucleases are responsible for the degradation. EDTA has been used as an additive to urine in some, but not all of the cancer studies of TR-ctDNA to date. Furthermore, the final concentrations of EDTA used have been variable, and could contribute to variation across studies. Additional approaches for urine DNA stabilization are also being developed (P. Li et al. 2019). Additional rigorous studies of methods and protocols for urine collection, cell-free DNA stabilization and specimen processing for TR-ctDNA analysis are an essential next step for clinical development of TR-ctDNA-based biomarkers.

Other pre-analytical variables include ones that are more biological in nature, such as the optimal time of day of urine collection, impact of comorbidities such as kidney disease, the impact of medications including certain chemotherapy agents that may be nephrotoxic, as well as variation in hydration status and use of diuretics. Such variables have begun to be examined in the context of TR-ctDNA (Augustus et al. 2020) and assessing their potential impact in specific cancer types may be important to study to increase reliability and consistency in TR-ctDNA assays. It is also not known yet whether approaches that correct for biologically-based, pre-analytic variations such as sample-to-sample variations in glomerular filtration will be of any utility. It may also be possible that much of the confounding effect of biological variations could be overcome by collecting pooled urine samples from a patient over a few days, to average out the impact of factors such as day-to-day variation in glomerular filtration, hydration, etc.

An additional area of research and development is defining the optimal methods and assays for TR-ctDNA biomarker analysis. This includes methods for the initial processing to obtain cell-free urine, extraction of cell-free DNA, and technologies for quantifying specific biomarkers such as mutant or methylated TR-ctDNA fragments. It appears to be important that optimized urine centrifugation protocols are used to prevent contamination with genomic DNA found in cells in the urine (Augustus et al. 2020). Extraction of cell-free DNA from urine can be challenging, in particular from larger volumes (e.g., >10 ml) of urine. Protocols for efficient cfDNA from urine samples of greater volumes are beginning to be developed (Zainabadi et al. 2019), and will benefit from more widespread validation and standardization across studies. Methods for analysis of TR-ctDNA biomarkers are varied, but two very common approaches are droplet digital PCR (ddPCR)-based assays and NGS. The ddPCR approach has the advantage of high sensitivity and reproducibility, high sample throughput and relatively quick turnaround time, and lower cost compared to NGS. However, ddPCR has limited multiplexing capability compared to NGS-based methods and therefore NGS may be preferable in settings where assay of a large number of DNA targets is desirable. It has been reported that the adoption of NGS-based technology reduces the need for laboratory personnel time dedicated to testing activities, which can reduce overall cost of testing per sample (Pisapia et al. 2022). Thus, in appropriate settings, NGS technologies can provide a practical and robust, cost-saving solution for routine analysis (Pisapia et al. 2022; Malapelle et al. 2021).

While there are important gaps in knowledge to be addressed as part of the development process to translate TR-ctDNA tests into the clinic, the recent advances in this field are exciting because they open up opportunities for completely non-invasive ctDNA testing using urine for a broad range of cancer types. Table 1 highlights key TR-ctDNA studies in oncology published thus far, as well as non-oncologic studies that have helped develop the concept of TR-ctDNA. This presents exciting opportunities to advance patient care and clinical research, with myriad potential clinical applications. In addition to applications demonstrated for blood-based ctDNA assays (e.g., tumor genotyping, detecting and monitoring of treatment responses, minimal residual disease detection, and early detection of primary cancer or recurrence), the ability to monitor ctDNA kinetics at high time-resolution (Husain et al. 2017) is uniquely enabled by TR-ctDNA. Furthermore, given that urine can be collected in much larger volumes than blood, it may be possible to increase clinical sensitivity, especially for detection of smaller, early-stage cancers, simply through analysis of large urine volumes. This hypothesis will be important to test in future studies. As understanding and control of pre-analytical variables increases and methods become standardized, this will pave the way for clinical biomarker validation studies in large patient populations to better characterize biomarker performance, which is an essential part of the development process toward clinical translation.

**TABLE 1:** Key Trans-Renal cell-free DNA Studies in Non-Oncology and Oncology Contexts.

Non-oncology key studies in trans-renal DNA	
Author (year)	Key Finding(s)
Title	
Botezatu et al. (2000)	First demonstration of transrenal cell-free DNA (cfDNA) in both animal and human models; Proof-of-concept that the renal barrier in both mice and humans is permeable to cfDNA
Genetic analysis of DNA excreted in urine: a new approach for detecting specific genomic DNA sequences from cells dying in an organism	
Koide et al. (2005)	Demonstrated that maternal urine may be useful for detection of fetal DNA; Fetal DNA in urine is more fragmented than that found in plasma
Fragmentation of cell-free fetal DNA in plasma and urine of pregnant women	
Cannas et al. (2008)	Small *M. tuberculosis* DNA fragments can be detected in urine of patients with pulmonary *tuberculosis*, suggesting that transrenal detection of *M.* tuberculosis infection may be feasible
Mycobacterium *tuberculosis* DNA detection in soluble fraction of urine from pulmonary *tuberculosis* patients	
Hung et al. (2009)	First to demonstrate the presence of donor-derived DNA in urine of hematopoietic stem cell transplant recipients
Presence of donor-derived DNA and cells in the urine of sex-mismatched hematopoietic stem cell transplant recipients: implication for the transrenal hypothesis	
Shekhtman et al. (2009)	Single-copy fetal DNA sequences can be detected in the urine of pregnant women using adequate methods for DNA isolation and analysis
Optimization of transrenal DNA analysis: detection of fetal DNA in maternal urine	
Tsui et al. (2012)	Confirmed the existence of and characterized transrenal fetal DNA in maternal urine using massively parallel sequencing
High resolution size analysis of fetal DNA in the urine of pregnant women by paired-end massively parallel sequencing	
Yu et al. (2013)	Massively parallel sequencing of maternal plasma and urinary DNA allows for high-resolution study of clearance profiles of circulating fetal DNA
High-resolution profiling of fetal DNA clearance from maternal plasma by massively parallel sequencing	
Hyman et al. (2015)	Urine analysis of cfDNA *BRAF V600E* mutations in urine provides a reliable method of detecting mutation status and may be useful as a biomarker to monitor response to treatment in Langerhans Cell Histiocytosis and Erdheim-Chester Disease
Prospective Blinded Study of BRAFV600E Mutation Detection in Cell-Free DNA of Patients with Systemic Histiocytic Disorders	
Cheng et al. (2017)	Characterized the composition, half-life, and variation in origins of urine cfDNA using genome-wide bisulfite sequencing
Genomewide bisulfite sequencing reveals the origin and time-dependent fragmentation of urinary cfDNA	

Oncology key studies in trans-renal ctDNA		
Author (year)	Cancer type	Key finding(s)
Title		
Eisenberger et al. (1999)	Renal	Microsatellite DNA analysis of urine in patients with renal cancer showed high concordance with microsatellite changes found in primary tumor; First study to indicate that microsatellite DNA analysis of urine could provide a tool for detection of resectable kidney cancer
Diagnosis of renal cancer by molecular urinalysis		
Su et al. (2004)	Colorectal	Provided first evidence for detection of KRAS mutant DNA in urine of a patient with colorectal cancer
Human Urine Contains Small, 150 to 250 Nucleotide-Sized, Soluble DNA Derived from the Circulation and May Be Useful in the Detection of Colorectal Cancer		
Su et al. (2005)	Colorectal	Detection of KRAS mutation in urinary DNA of patients with colorectal carcinoma shows high concordance with disease tissue
Detection of a K-ras mutation in urine of patients with colorectal cancer		
Su et al. (2008)	Colorectal	Initial evidence that KRAS mutation might be detectable in some patients with pre-cancerous lesions (i.e., adenomatous colorectal polyps)
Detection of Mutated K-ras DNA in Urine, Plasma, and Serum of Patients with Colorectal Carcinoma or Adenomatous Polyps		
Chan et al. (2008)	Nasopharyngeal	Circulating Epstein-Barr virus DNA from nasopharyngeal carcinoma (NPC) can be trans-renally excreted into urine
Quantitative analysis of the transrenal excretion of circulating EBV DNA in nasopharyngeal carcinoma patients		
Song et al. (2012)	Colorectal	A colorectal cancer-associated methylated DNA marker (hypermethylated vimentin gene) can be detected, presumably as trans-renal DNA, in urine of patients with colorectal cancer
Detection of hypermethylated vimentin in urine of patients with colorectal cancer		
Berz et al. (2015)	Non-Small Cell Lung Cancer	Early case study demonstrating the detectability of the EGFR T790M therapeutic resistance mutation in urine as TR-ctDNA
Non-Invasive Urine Testing of EGFR Activating Mutation and T790M Resistance Mutation in Non-Small Cell Lung Cancer		
Reckamp et al. (2016)	Non-Small Cell Lung Cancer	EGFR mutant DNA derived from non-small cell lung cancer (NSCLC) tumors can be detected in urine and plasma
A Highly Sensitive and Quantitative Test Platform for Detection of NSCLC EGFR Mutations in Urine and Plasma		
Husain et al. (2017)	Non-Small Cell Lung Cancer	Dynamic changes in EGFR activating and resistance mutation levels detected in urine are associated with tumor response within days of therapy for NSCLC patients receiving anti-EGFR tyrosine kinase inhibitors; Daily sampling of circulating tumor DNA (ctDNA) has potential for early assessment of treatment response
Monitoring Daily Dynamics of Early Tumor Response to Targeted Therapy by Detecting Circulating Tumor DNA in Urine		
Sands et al. (2017)	Non-Small Cell Lung Cancer	Evidence that urine ctDNA detection could be cost-effective and help patients avoid tissue biopsies and associated complications
Urine circulating-tumor DNA (ctDNA) detection of acquired EGFR T790M mutation in non-small-cell lung cancer: An outcomes and total cost-of-care analysis		
Hann et al. (2017)	Hepatocellular	Evidence for urine TR-ctDNA in hepatocellular cancer patients
Detection of urine DNA markers for monitoring recurrent hepatocellular carcinoma		
Shi et al. (2017)	Gastric	Evidence that EGFR mutant TR-ctDNA is detectable in urine of gastric cancer patients and shows strong concordance with plasma ctDNA profiles
Dynamic tracing for epidermal growth factor receptor mutations in urinary circulating DNA in gastric cancer patients		
Li et al. (2017)	Non-Small Cell Lung Cancer	Demonstrated detection of trans-renal DNA in non-small cell lung cancer patients and characterized concordance with plasma ctDNA and tissue mutation status in early and late stages of cancer
Utility of urinary circulating tumor DNA for EGFR mutation detection in different stages of non-small cell lung cancer patients		
Chen et al. (2017)	Non-Small Cell Lung Cancer	Urinary ctDNA analysis showed close agreement on EGFR mutation status when compared to primary tissue in patients with NSCLC; Analysis of urinary cfDNA at different time points showed correlation to treatment efficacy
Urinary circulating DNA detection for dynamic tracking of EGFR mutations for NSCLC patients treated with EGFR-TKIs		
Wang et al. (2017)	Non-Small Cell Lung Cancer	Longitudinal monitoring showed an increase in quantity of TR-ctDNA which was associated with disease progression
Investigation of transrenal KRAS mutation in late stage NSCLC patients correlates to disease progression		
Liu and Liu (2018)	Breast	Urinary TR-ctDNA analysis in breast cancer patients associated with relapse after surgery
Association of urinary and plasma DNA in early breast cancer patients and its links to disease relapse		
Hu et al. (2018)	Non-Small Cell Lung Cancer	Urinary TR-ctDNA levels after treatment associated with frequency of recurrence
Urinary circulating DNA profiling in non-small cell lung cancer patients following treatment shows prognostic potential		
Zhang et al. (2018)	Non-Small Cell Lung Cancer	Measurement of EGFR mutations in NSCLC patients using plasma and urine TR-ctDNA analysis showed concordance with tumor tissue mutation analysis
Comparison of circulating DNA from plasma and urine for EGFR mutations in NSCLC patients		
Crisafulli et al. (2019)	Colorectal	Demonstrated feasibility of analysis of trans-renal DNA in metastatic colorectal cancer patients using whole exome sequencing
Whole exome sequencing analysis of urine trans-renal tumour DNA in metastatic colorectal cancer patients		
Terasawa et al. (2019)	Pancreatic ductal adenocarcinoma	Provided evidence for feasibility of urine TR-ctDNA detection in patients with pancreatic ductal adenocarcinoma; showed preliminary evidence that glomerular filtration rate may correlate with clinical sensitivity of urine TR-ctDNA-based assay
Utility of liquid biopsy using urine in patients with pancreatic ductal adenocarcinoma		
Yu et al. (2020)	Colorectal	Urine TR-ctDNA shows high concordance with plasma ctDNA and tumor tissue mutations and changes over time may be associated with disease progression
Circulating tumor cell free DNA from plasma and urine in the clinical management of colorectal cancer		
Guan et al. (2020)	Breast	Higher urine TR-ctDNA levels at 6-months post-treatment may be associated with greater risk of relapse in breast cancer patients
Utility of urinary ctDNA to monitoring minimal residual disease in early breast cancer patients		
Smith et al. (2020)	Renal	Evidence that urine ctDNA may capture tumor heterogeneity in kidney cancer
Comprehensive characterization of cell-free tumor DNA in plasma and urine of patients with renal tumors		
Nuzzo et al. (2020)	Renal	Showed the validity of a cell-free methylated DNA immunoprecipitation and high throughput sequencing assay to detect renal cell carcinoma based on urine cfDNA
Detection of renal cell carcinoma using plasma and urine cell-free DNA methylomes		
Bach et al. (2021)	Colorectal	A TR-ctDNA marker panel based on detecting methylated DNA markers showed 70% sensitivity with 86% specificity for colorectal cancer detection
Detection of colorectal cancer in urine using DNA methylation analysis		
Ohta et al. (2021)	Colorectal	Provided evidence for increased clinical sensitivity of colorectal cancer detection when urine TR-ctDNA and plasma ctDNA assays are combined
Detection of KRAS mutations in circulating tumour DNA from plasma and urine of patients with colorectal cancer		
Pellini et al. (2021)	Colorectal	Presented evidence for feasibility of urine TR-ctDNA-based minimal residual disease detection in patients with oligometastatic colorectal cancer
ctDNA MRD Detection and Personalized Oncogenomic Analysis in Oligometastatic Colorectal Cancer From Plasma and Urine		
Mouliere et al. (2021)	Glioma	Used a targeted hybridization-capture next-generation sequencing approach to detect trans-renal ctDNA in patients with glioma
Fragmentation patterns and personalized sequencing of cell-free DNA in urine and plasma of glioma patients		

The fact that TR-ctDNA testing involves a completely non-invasive, self-collected sample that can be mailed to a central laboratory is also relevant to development toward the clinic. Multiple studies have shown that patients prefer non-invasive testing options, such as saliva and urine, when possible, as compared to blood testing (Dhima et al. 2013; Koka et al. 2008; Osborne et al. 2018). In light of the COVID-19 pandemic, recent work has also illuminated the importance of self-collected samples for increasing adherence to cancer screening (Gorin et al. 2021). These preferences for bodily fluid collection may influence willingness to participate in research trials and adherence to longitudinal cancer surveillance programs. As such, urine-based assays may improve access for underserved populations and those who cannot readily travel to hospitals and testing facilities. The ability to provide disease surveillance remotely, thus reducing the need for visits to clinical facilities, might also prove to reduce healthcare costs, even in settings where healthcare facility access is not a limiting factor. A recent cost-effectiveness analysis of urine TR-ctDNA for therapy resistance mutation detection in non-small cell lung cancer found a reduced overall cost-of-care using this approach (Sands, Li, and Hornberger 2017).

In conclusion, while much work remains to be done to bring TR-ctDNA testing into widespread clinical use, trans-renal cell-free DNA research has gained momentum over the past 2 decades. It represents an emerging liquid biopsy approach that has potential for broad clinical impact because of the completely non-invasive, self-collected sampling approach and the accumulating base of evidence demonstrating TR-ctDNA detection across diverse cancer types.
